# Optimizing the Structural, Electrical and Thermoelectric Properties of Antimony Telluride Thin Films Deposited on Aluminum Nitride-coated Stainless Steel Foil

**DOI:** 10.1038/s41598-020-63954-0

**Published:** 2020-04-24

**Authors:** Aziz Ahmed, Seungwoo Han

**Affiliations:** 1 Department of Nano-Mechatronics, Korea University of Science and Technology (UST), 217 Gajeong-ro, Yuseong-gu, Daejeon, 305-350 Republic of Korea; 2 Department of Nano-Mechanics, Korea Institute of Machinery and Materials (KIMM), 156 Gajeongbuk-ro, Yuseong-gu, Daejeon, 305-343 Republic of Korea

**Keywords:** Materials science, Materials for energy and catalysis

## Abstract

In this study, we examined the thermoelectric (TE) properties of co-evaporated p-type antimony telluride (Sb_2_Te_3_) thin films on aluminum nitride (AlN)-coated stainless steel foil substrates. We investigated the influence of composition and substrate temperature on the thin-film microstructure and transport properties, by varying the tellurium (Te) concentration in the thin films as well as the substrate temperature during deposition (room temperature (RT) and 300 °C). Thin films prepared with an RT substrate were further annealed at 264 °C to obtain crystallized thin films with high phase purity. Columnar thin films with large grains and a standard multi-oriented crystal structure were obtained when thin films were deposited on substrates heated to 300 °C. Thin films deposited at RT and subsequently annealed at 264 °C had a dense, layered microstructure, with a preferential c-axis or (00 *l*) texture as the compositions approached phase stoichiometry. The temperature dependence of the thermoelectric properties was measured, and variations were interpreted in terms of the deviation from stoichiometry and the obtained microstructure. A maximum power factor (PF) of 0.87 mW/m ∙ K^2^ was obtained for off-stoichiometric 65.0 at% Te thin film, which was the highest among the samples deposited at high substrate temperatures. A higher PF of 1.0 mW/m ∙ K^2^ was found for off-stoichiometric thin films with 64.5 at% Te, which was deposited at RT and subsequently annealed. The improvement of thermoelectric power in films containing excess Te could be related to energy dependent carrier scattering at the Sb_2_Te_3_/Te interface.

## Introduction

Thermoelectric (TE) materials can directly convert thermal energy into electrical energy through the Seebeck effect and electrical energy into thermal energy through the Peltier effect, without entailing any mechanical parts or moving fluids; thus, these material systems are clean, sustainable, and highly reliable. Efficient TE thin-film devices have numerous applications in the microelectronics industry, such as thermal management or spot cooling of various components in a microsystem. TE cooling efficiency and output power depend primarily on the properties of the material. These properties are specified collectively by the material’s figure of merit, ZT, through the relation ZT = (S^2^T/ρk), where *S* is the Seebeck coefficient, *ρ* is the electrical resistivity, *k* is the thermal conductivity, and *T* is the temperature in Kelvin. The commercial use of TE energy convertors has yet to become widespread, due to their relatively low energy conversion efficiency. Efforts are currently underway to improve the cost-effectiveness and efficiency of these devices, while reducing the scale of the device dimensions^1–6^.

Telluride (Te)-based compounds have been studied extensively over the past several decades due to their considerable ZT at room temperature (RT). Antimony telluride, Sb_2_Te_3_, is a V-IV binary compound with a narrow band gap; this compound has a rhombohedral structure with space group (..) and contains five atoms in each unit cell^7–12^. To improve solid-state power generation and cooling, TE materials in low-dimensional structures, such as thin films, superlattices, and quantum wires, have been investigated for phenomena related to size, interface, and quantum confinement effects^13–18^. Hicks and Dresselhaus^15^ predicted that low-dimensional nanostructuring would increase the density of states of confined carriers near the Fermi level, while simultaneously enhancing phonon scattering, thereby increasing the ZT. Venkatasubramanian et al.^17^. reported a record high TE efficiency of 2.4 for a p-type Bi_2_Te_3_/Sb_2_Te_3_ superlattice structure prepared by molecular beam epitaxy (MBE); a ZT value of 3 was also reported for an n-type PbSeTe/PbTe quantum dot superlattice^18^. Furthermore, TE properties are known to depend strongly on the microstructure and composition of the TE material. It is relatively easier to control and optimize material preparation conditions with low-dimensional fabrication processes; TE analysis is also much easier.

The development and optimization of TE generators have attracted much interest, due to the explosive growth in the demand for self-powered wearable mobile electronics and sensors^19–23^. Advances in planar thin-film technology have allowed for a large choice of substrates, including those suitable for use in thin-film, flexible TE devices. Depending on the heat flow direction, a TE device is classified as either planar or vertical^22^. It has been established that the use of a metallic high thermal conductivity substrate is necessary to minimize the temperature drop across the substrate leading to improved thermal management and efficiency in a TE device^24^. However, TE measurement of thin films deposited on metallic/conducting substrates is limited by the metallic substrate that creates an electric short-circuit during measurements^25^. In most reports, Sb_2_Te_3_ thin films were deposited on rigid substrates. Several studies have examined TE thin-film depositions on polyimide (PI) substrates^13,26–28^, which, although flexible, do not possess the high thermal conductivity necessary for high power density and uniformity in thin-film properties. Other issues include the degradation of the PI substrate at high processing temperatures^26^.

A key research area of micro-TE devices involves the application of semiconductor thin-film technology to TE thin-film fabrication to improve device performance. Several techniques have been reported for growing Sb_2_Te_3_ TE thin films, such as thermal evaporation^13,29–31^, sputtering^26–28,32–34^, flash evaporation^35^, electrochemical deposition^25,36,37^, pulse laser deposition^38^, metal-organic chemical vapor deposition^39^, and MBE^9,10,40^. However, some of these processes tend to be complicated, time-consuming, and require expensive precursors and equipment. Therefore, in this study, a thermal evaporation deposition method was selected, which offers the advantages of low-cost fabrication and a short processing time. This work reports on the optimal growth of p-type Sb_2_Te_3_ thin films on aluminum nitride (AlN)-coated stainless steel foil substrates by means of co-evaporation, to better understand and circumvent the ZT limitations. Detailed analyses of the film composition, microstructure, and transport properties were conducted in an attempt to optimize TE performance. A major challenge, especially when depositing thin films at higher substrate temperatures, is control over the phase stoichiometry, which frequently leads to the formation of defects in the thin films^9,36^. These defects significantly influence the thin-film carrier scattering mechanisms and consequently the transport properties, so it is important to control the defect concentration for optimal device performance. Yoo *et al*. ^37^ obtained a high Seebeck coefficient for Sb_2_Te_3_ thin films, prepared using electro-deposition process, due to the formation of nanodots of Te atoms in the samples. Similarly, Zhang *et al*.^40^ reported a high Seebeck coefficient and a high power factor for MBE deposited Sb_2_Te_3_/Te multilayer thin films. This work therefore specifically focused on the measurement of transport properties as a function of film composition, in which other parameters such as the substrate type, substrate temperature, and film thickness remained constant. The observed variations in the transport properties were interpreted and special emphasis was placed on the carrier energy filtering effect which is regarded as an attractive method to improve the Seebeck coefficient^41,42^. The proposed method can be used to fabricate efficient Sb_2_Te_3_ thin films without any intentional doping and with the added possibility of extending the technology to include flexible TE devices.

## Methods

To ensure structure uniformity and reproducibility of the transport properties, the stainless steel foils underwent a thorough polishing process. Square foil samples (side dimension: 20 mm; thickness: 500 µm) were polished with sand paper and a diamond and silica suspension, followed by cleaning with alcohol and water. The foil thickness after polishing was 250 µm; further thinning was difficult, due to the limitations imposed by the employed polishing apparatus.

AlN thin films were grown on a polished stainless steel substrate, using a direct current (DC) reactive sputtering system (ATC Orion Series, AJA International Inc., Scituate, MA, USA). A high-purity Al (99.999% pure) target (diameter: 2 inches) was used. All substrates were cleaned ultrasonically prior to deposition using isopropanol, acetone, ethanol, and deionized water, and dried with flowing nitrogen. The chamber was evacuated to a vacuum pressure of 5 × 10^−7^ Torr prior to deposition. The substrate was kept at 300 °C and RT during deposition, respectively, for subsequent TE thin-film experiments conducted at high and low substrate temperatures. AlN thin films deposited at RT were further annealed at 300 °C for 3 hours prior to the thermoelectric thin film deposition. The sample was rotated at 10 rpm to ensure uniformity in film thickness and composition. The DC power applied to the Al target was 100 W and the deposition time was 3 h. During the experiment, the working pressure was maintained at 8 mTorr using a mixture of 5 sccm of argon and 7 sccm of nitrogen flowing in the chamber as the sputtering gas.

In this work, Sb-Te films were deposited by thermal co-evaporation of Sb and Te evaporants using an effusion cell evaporator system (Alpha Plus Co., Ltd., Gyeongsangbuk-do, Korea). Evaporation tubes containing high-purity (99.999%) Sb and Te elemental evaporants were placed in separate heater assemblies, which were connected to independent power supplies. The evaporation rates of each source were adjusted individually via two power controllers and monitored by two quartz crystal sensors. Deposition was performed after evacuating the evaporation chamber to a low vacuum pressure of 3 × 10^−7^ Torr, and the substrate was rotated to ensure uniformity in film composition and thickness. The compositions of the as-deposited thin films were varied by adjusting the individual source deposition rate. The deposition rate of Sb was fixed at 1.5 Å/s; the deposition rate for Te was varied over the range of 3.5–4.5 Å/s for experiments performed at a high substrate temperature of 300 °C. These samples were further annealed for 3 h at 220 °C inside the vacuum chamber after deposition. For experiments in which the substrate was held at RT during deposition, the Sb deposition rate was fixed at 1.5 Å/s, whereas the deposition rate for Te was varied from 2.5 to 4.0 Å/s with further post-deposition annealing at 264 °C for 1 h inside the vacuum chamber.

To determine the optimized film deposition parameters, the influences of composition and substrate temperature on crystal quality and orientation, microstructure, and TE properties were investigated using high-resolution scanning electron microscopy (HR-SEM), energy dispersive X-ray spectroscopy (EDS), and X-ray diffraction (XRD). The details of the characterization process are described in our previous publications^14,43^. The Hall measurements were performed on several mm side square samples using an Ecopia HMS-5000 room temperature Hall effect system. Few samples could be prepared for analysis due to certain difficulties and hall measurement data is discussed qualitatively in this study. The in-plane electrical and TE properties were measured using a commercially available system (TFTEP-800, SeePel Co. Ltd., Gunpo, Korea). The Seebeck coefficient was determined as the ratio of the Seebeck voltage (ΔV) across the films with respect to the temperature difference (ΔT). For this purpose, two opposite ends of the samples were thermally connected to two heaters maintained at different temperatures. A four-point probe method was used to determine the electrical resistivity of the samples. The power factor PF, given by S^2^/ρ, was calculated using the measured values of the electrical resistivity and the Seebeck coefficient. Measurement errors for PF remained <10% in all studied cases.

## Results and Discussion

Series of Sb-Te thin films with different compositions were prepared at two substrate temperatures, to determine the main factors influencing the TE properties. Measurement complications associated with the use of a conducting foil substrate were minimized by depositing the films on stainless steel foils pre-coated with an AlN electrical insulation layer. AlN was selected as the insulation layer^44^, due to its high thermal conductivity^45^, which effectively minimizes the thermal resistance of the substrate assembly specifically for a cross-plane TE device configuration. In this study, we focused only on the electrical properties of the insulation layer. The extra thickness of the foil also ensured good thermal contact between the substrate and the heater during thin film deposition and annealing. In this regard, although substrate flexibility is compromised to some extent, a sufficient foil thickness ensures microstructure uniformity and measurement reliability of the transport properties over the entire thin-film surface.

To obtain thin films with different compositions, we varied the Te evaporation rate while keeping the deposition rate for Sb constant. To minimize measurement error, film compositions were determined over distinct areas of the sample surface, with only averaged values reported. Hereafter, the films are identified by their Te content (at% composition). The thickness of all of the deposited thin films ranged from 1–2 μm. Tables 1 and 2 shows the nominal compositions and actual compositions of the thin films prepared on substrates kept (during deposition) at high temperature and room temperature respectively, after the thermal annealing steps.

**Table 1:** The nominal compositions and actual compositions of the Sb-Te thin films fabricated at high substrate temperatures.

Nominal Composition	Actual Composition	Identified Te content (at. %)
Sb_2_Te_3+x_/x = 0	Sb_2_Te_3.04_	60.3
Sb_2_Te_3+x_/x = 0.7	Sb_2_Te_3.72_	65.0
Sb_2_Te_3+x_/x = 0.2	Sb_2_Te_3.26_	62.0
Sb_2_Te_3+x_/x = 0.4	Sb_2_Te_3.42_	63.1
Sb_2_Te_3+x_/x = 0.6	Sb_2_Te_3.6_	64.3
Sb_2_Te_3+x_/x = 0.7	Sb_2_Te_3.74_	65.2

**Table 2:** The nominal compositions and actual compositions of the annealed Sb-Te thin films.

Nominal Composition	Actual Composition	Identified Te content (at. %)
Sb_2_Te_3+x_/x = −0.1	Sb_2_Te_2.86_	58.9
Sb_2_Te_3+x_/x = 0.3	Sb_2_Te_3.26_	62.0
Sb_2_Te_3+x_/x = 0.6	Sb_2_Te_3.64_	64.5
Sb_2_Te_3+x_/x = 0.9	Sb_2_Te_3.94_	66.3
Sb_2_Te_3+x_/x = 1.8	Sb_2_Te_4.76_	70.4
Sb_2_Te_3+x_/x = 2.5	Sb_2_Te_5.52_	73.4

### Structural and thermoelectric properties of thin films fabricated at high substrate temperatures

The thin films fabricated on a substrate held at 300 °C during deposition underwent an additional annealing treatment at 220 °C under high vacuum for 3 h. This annealing step carried out at a temperature which was significantly lower than the deposition temperature enhanced the transport properties slightly by virtue of reduced thin film defects while preventing any Te loss through sublimation. The experimental data for film composition, structure and thermoelectric properties presented in this section were measured after the aforementioned annealing step. At high substrate temperatures during deposition, the re-evaporation of Te from the deposited film can become an issue. Thus, high Te evaporation rates were used to deposit thin films with excess Te content. Some samples were prepared by depositing a 20-nm-thick layer of chromium (Cr) metal as a thermal shock resistant adhesion layer prior to the deposition of the TE films^34^; the effects of the adhesion layer on the TE properties of the films were evaluated.

**Figure 1:**
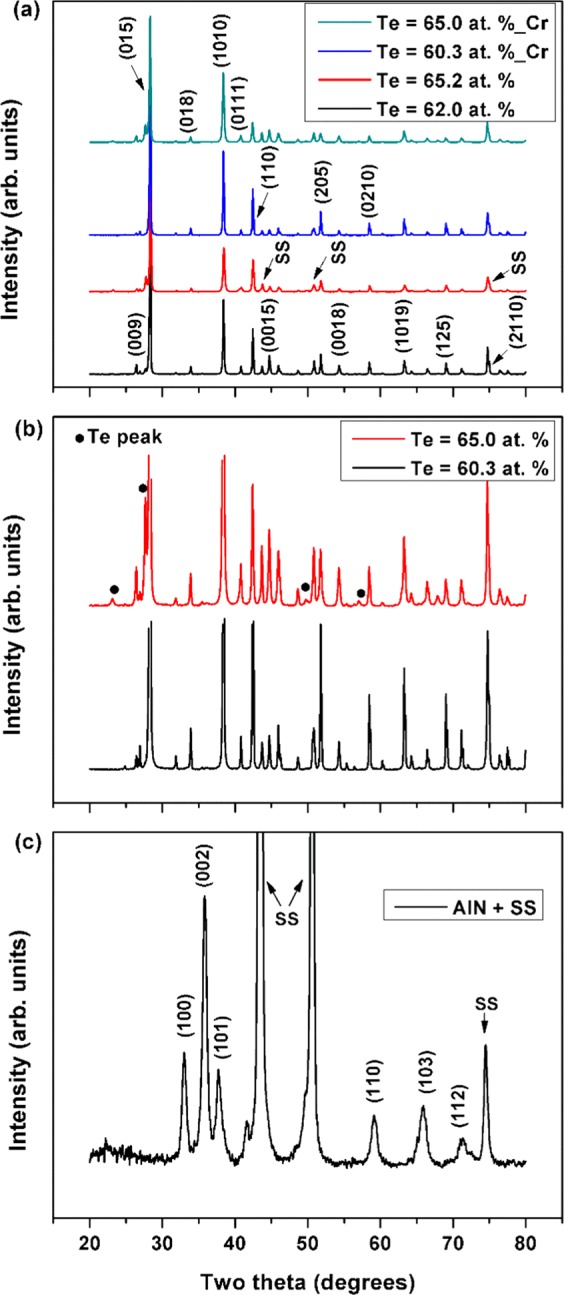
X-ray diffraction (XRD) patterns for (**a**) antimony-tellurium (Sb-Te) thin films deposited at high substrate temperatures, (**b**) Sb-Te thin films in which high-intensity Sb-Te peaks were removed for better visualization of the additional less-intense Te peaks observed due to deviation from the film stoichiometry, and (**c**) aluminum nitride (AlN) thin film deposited on stainless steel foil.

Figure 1a presents the XRD patterns of selected Sb-Te thin films that show the polycrystalline nature of the prepared samples. The (hkl)-plane indices and peak intensities obtained were in good agreement with the standard powder diffraction patterns for Sb_2_Te_3_. The XRD peaks were indexed as the rhombohedral Sb_2_Te_3_ phase; the (015) plane peak was the most intense. Other high intensity peaks, including those corresponding to (1010) and (110) plane, were also observed, indicating a standard, multi-oriented Sb_2_Te_3_ crystal structure in our thin-film samples. For thin films deposited at high substrate temperatures, XRD peaks of significant intensities could not be observed between Two-Theta values of 5° and 20° which was consistent with the standard Sb_2_Te_3_ diffraction patterns (JCPDS-015-0874). The Two-Theta range in Fig. 1 was therefore selected in such a way as to ensure that Sb_2_Te_3_, Te as well as substrate peaks of substantial intensities could be clearly observed and easily distinguished. Due to significant deviations from phase stoichiometry, additional XRD peaks representing elemental Te were detected in significantly off-stoichiometric samples. In Fig. 1b, the high-intensity XRD peak data for Sb_2_Te_3_ were removed to better illustrate the positions and intensities of the Te peaks. The intensity of Te peaks depended on its relative concentration in the samples. Apart from the additional Te peaks and relative differences in peak intensities, no other significant changes in the XRD patterns were observed when the Te content in the thin film increased; thus, the preferred film orientation was preserved. The intensity, sharpness, and narrow widths of XRD peaks indicated that the thin films had a high crystalline quality. In our XRD patterns for TE films, we did not clearly observe any peaks that represented the underlying AlN thin-film layer. A separate XRD experiment was performed for AlN thin film deposited on a bare stainless steel substrate; the results are shown in Fig. 1c. The (002) plane peak was observed to be the most intense, and the presence of several AlN peaks indicated a significant degree of polycrystallinity in the insulation layer.

**Figure 2:**
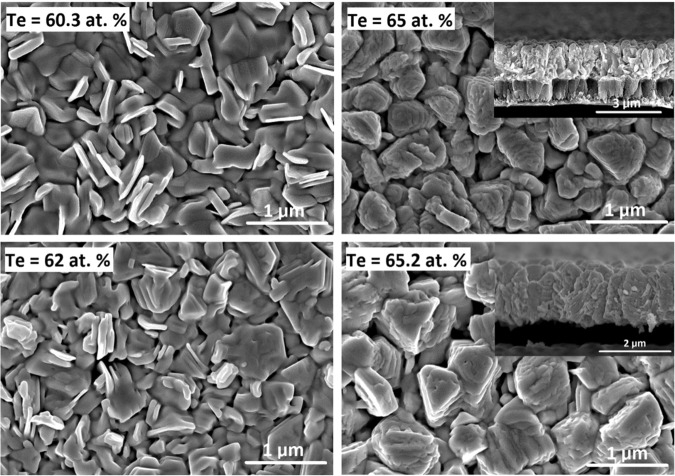
Scanning electron microscopy (SEM) images showing the surface and (inset) cross-sectional morphologies of Sb-Te thin films prepared at high substrate temperatures.

Figure 2 presents the surface and cross-sectional images of the selected thin films. All samples, regardless of composition, had a homogeneously distributed and well-defined polycrystalline grain structure with large-sized grains, corresponding to a continuous, well-crystallized thin film for the given deposition conditions. A polycrystalline structure with large grains and intervening voids can be explained in terms of a high substrate temperature. At higher temperatures, the substrate imparts a higher kinetic energy to the deposited atoms. This, in turn, reduces the diffusion time for these atoms to move to proper lattice growth points and results in the rapid formation of large multi-oriented grains via the coalescence of smaller atoms and grains. Cross-sectional SEM images (Fig. 2) show columnar-shaped structure grown along its preferred growth direction, which appears to be somewhat perpendicular to the substrate surface. This indicates that film nucleation and growth in the vertical direction is faster than in the horizontal direction of the plane. The AlN microstructure comprised tightly packed columns grown perpendicular with respect to the substrate surface. The adhesion between the TE thin film and insulation layer was remarkably enhanced by the introduction of a Cr adhesion layer, as detachment did not occur while preparing the sample for SEM cross-sectional analysis.

**Figure 3:**
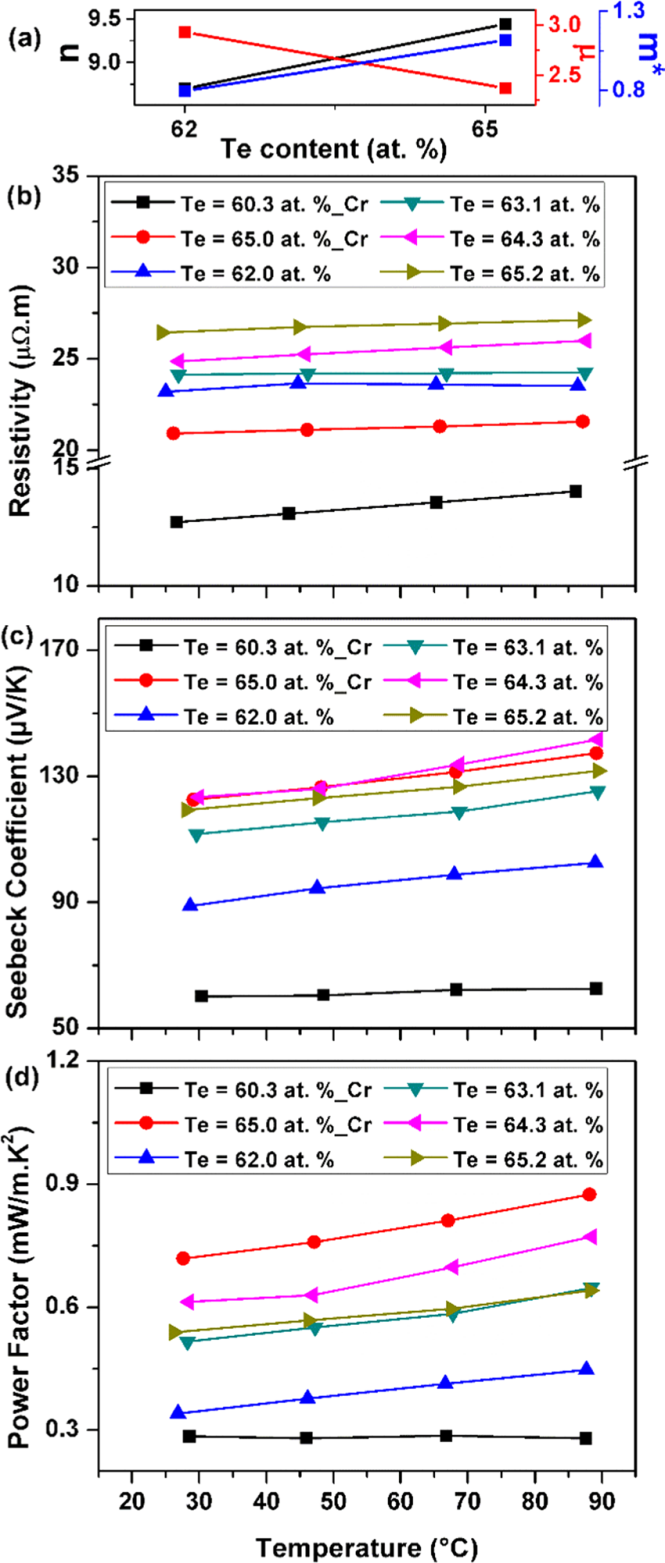
(**a**) Carrier concentration (10^25^ m^−3^), mobility (10^−3^ m^2^/V.s) and density-of-state (DOS) effective mass (10^−30^ kg) of the Sb-Te thin films as a function of Te content. Temperature variation of the (**b)** electrical resistivity, (**c**) Seebeck coefficient, and (**d**) power factor (PF) of the Sb-Te thin films prepared at high substrate temperatures.

Figure 3a present the carrier concentration *n*, mobility *µ* and calculated density-of-state DOS effective mass m* (discussed later in the manuscript) as a function of Te content whereas Fig. 3b present the temperature dependence of the electrical resistivity, given by ρ = 1/neµ, where *e* is the electron charge, for thin films deposited with and without a Cr adhesion layer. The thin films with compositions closer to phase stoichiometry had lower electrical resistivity. The electrical resistivity of the thin film increased gradually with Te content, which is consistent with the findings of previous studies^13,29,40^. The electrical resistivity of the sample increased with the temperature in most cases. The observed linear change in the resistivity with temperature indicates the phase purity of the thin films. The hall mobility decreases upon increasing the Te content of the films while the carrier concentration increases slightly.

One way to explain this behavior is by considering that excess Te in off-stoichiometric samples, as confirmed by the XRD analysis, is randomly dispersed in the host thin films in elemental form as a second phase. This excess Te may create a Sb_2_Te_3_/Te interface where a potential barrier is formed. The observed decrease in carrier mobility could then be directly related to the carrier scattering at the interface through the following relation presented by Savchuk^46^:

1 $${\mu }_{i} \sim {({m}^{\ast })}^{-1/2}{N}_{i}^{-1}{T}^{3/2}$$

where *N*_*i*_ is the ionized impurity density. The electrical resistivity increases with Te content because the strong decrease of carrier mobility, due to the inability of low energy carriers to overcome the potential barrier, was not compensated by the slight increase in carrier concentration. The magnitude of electrical resistivity obtained suggests that the deposition temperature was sufficiently high to produce the necessary crystallization, while minimizing excessive re-evaporation of Te. The electrical resistivities of the thin films prepared without a Cr layer were higher than those prepared with the adhesion layer, which was consistent with other reports^34^.

Figure 3c present the temperature variation of the Seebeck coefficient for thin films deposited with and without a Cr adhesion layer. The Seebeck coefficient for the thin films was positive, which indicates that the films are p-type; the value for the coefficient increased gradually with the Te content of the thin film, beyond that for phase stoichiometry. This finding can be explained in terms of the theoretical model for energy filtering of charge carriers at the Sb_2_Te_3_/Te interface. Faleev and Leonard^42^ explained in detail that the carrier scattering regimes as well as the overall underlying physics related with metal atoms dispersed in a host material is quite different from the one that involves other structural defects (such as point defects). The buildup of a potential barrier due to electronic band bending at the interface ensures that only the carriers with energy higher than the barrier height can pass through whereas carriers with lower or insufficient energy are effectively scattered. This energy dependent carrier scattering, i.e. the carrier energy filtering effect, leads to the enhancement of the Seebeck coefficient. The aforementioned argument could be proved by the Mott relation given by the following equation^47^:

2 $$S=\frac{{\pi }^{2}{k}_{B}^{2}}{3e}T{\left\{\frac{d[ln(\sigma (E))]}{dE}\right\}}_{E={E}_{F}}$$

3 $$\sigma =ne\mu =\frac{n{e}^{2}\tau }{{m}^{\ast }}$$

where *k*_*B*_ is the Boltzmann constant, *σ* is the electrical conductivity, *τ* is the carrier relaxation time. The relaxation time is defined as the average flight time of charge carriers between successive scattering events. Due to the larger number of carrier scattering events taking place at the Sb_2_Te_3_/Te interface, the carrier relaxation time and mobility are affected and their dependence on the carrier energy increases significantly. Consequently for thin films with excess Te, the Seebeck coefficient is improved due to its strong dependence on the energy derivative of the relaxation time i.e. d (lnτ(E))/dE at the Fermi level energy as observed from the aforementioned relations. This indicates that perhaps deviation from stoichiometry may be good in the case of Sb-Te thin films. In all cases, the Seebeck coefficient increased with temperature. The thin film with 64.3 at% Te content and devoid of a Cr adhesion layer had the highest Seebeck coefficient value, of about 142 μV/K at 90 °C, among all cases studied. This was closely followed by a Seebeck coefficient of about 138 μV/K that we measured at 90 °C for the 65.0 at% Te film prepared with a Cr adhesion layer.

Assuming a single parabolic band, a simple electron transport model could also be used to describe the Seebeck coefficient of the thin films according to the following relation^12^:

4 $$S=\frac{8{\pi }^{2}{k}_{B}^{2}T}{3e{h}^{2}}{\left(\frac{\pi }{3n}\right)}^{2/3}{m}^{\ast }$$

where *h* is the Planck’s constant. Therefore, the Seebeck coefficient depends on both the carrier concentration and DOS effective mass. The above relation was used to calculate DOS effective mass of carriers using the measured values of carrier concentration and Seebeck coefficient at room temperature (Fig. 3a). The effective mass value increases with increasing Te content in the thin film samples which could be considered as additional evidence of the occurrence of the energy dependent carrier scattering or carrier energy filtering effect in off-stoichiometric samples leading to an enhancement of the Seebeck coefficient and a decrease in carrier mobility. Therefore, the tailoring of the Te content in the Sb_2_Te_3_ thin films can be a key to optimize its thermoelectric properties.

The PF of the thin films was calculated by dividing the square of the Seebeck coefficient by the electrical resistivity. Figure 3d present the PF and its temperature variations for thin films prepared with and without a Cr adhesion layer. The PF generally increased with temperature, as well as with Te content. Due to the quadratic dependence of the PF on the Seebeck coefficient, the thin films with a larger Seebeck coefficient generally had a higher PF. Among the thin films prepared without a Cr adhesion layer, the highest PF value of 0.77 mW/m ∙ K^2^ was obtained for the 64.3 at% Te thin film sample. An even larger PF of 0.87 mW/m ∙ K^2^ was noted for film with a Te content of 65.0 at% prepared with a Cr adhesion layer. Although the Seebeck coefficient in the latter case was not the largest, its relatively lower electrical resistivity was responsible for its better performance. One conclusion that can be drawn from these results is that the TE properties of Sb_2_Te_3_ thin films depend strongly on the film composition. Thus, the TE PF can be optimized by controlling the defect concentration in the form of excess Te atoms. We observed a meaningfully higher maximum PF for the off-stoichiometric thin film with a Cr adhesion layer. Lastly, the observed changes in the thin film TE properties are expected to be accompanied by significant changes in the Fermi level position, as well as the overall band structure.

### Structural and thermoelectric properties of annealed thin films pre-fabricated using substrates held at room temperature

Retaining a low substrate temperature during film deposition is advantageous, because the thin films synthesized at low temperature usually have a low surface roughness, and are therefore compatible with device fabrication processes. The as-deposited samples were amorphous. Further annealing at 264 °C under high vacuum for 1 h promoted crystallization and the phase purity of the thin films beside causing a reduction in the defect concentration. The annealing temperature was carefully chosen so as not to alter the composition of the thin films obtained in the as-deposited state, because Te can re-evaporate from the thin films at higher annealing temperatures. The compositions of the films listed in this section are re-measured values after thermal annealing.

**Figure 4:**
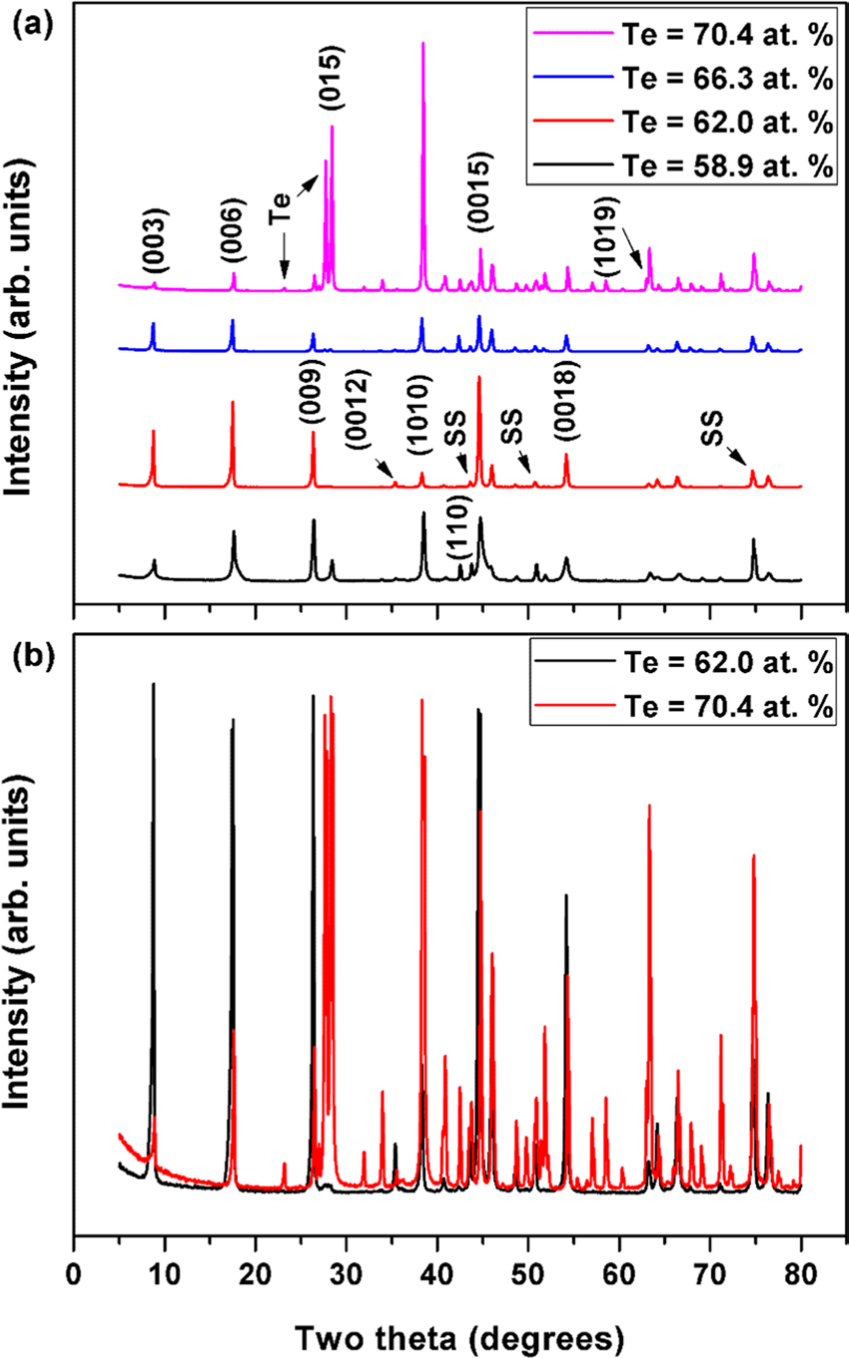
Room-temperature XRD patterns for (**a**) Sb-Te thin films after thermal annealing and (**b**) Sb-Te thin films after thermal annealing in which high-intensity Sb-Te peaks were removed for better visualization of the relative differences in XRD peak intensities due to deviation from the film stoichiometry.

Figure 4a,b presents the XRD patterns of the selected Sb-Te thin films after annealing. The patterns clearly illustrate the effects of substrate temperature (and the annealing method that followed) on the nucleation and crystal growth processes of the resulting thin films. Preferentially c-axis-oriented thin films were obtained when the compositions were stoichiometric or had smaller deviations. The strong and sharp diffraction peaks (003), (006), (009), (0015), and (0018) that correspond to strong reflections from (00 *l*) crystal planes are clearly evident in these samples. The intensity of (00 *l*)-oriented peaks was strong, compared with the standard powder diffraction patterns, and decreased significantly with large deviations from the phase stoichiometry. Other Sb_2_Te_3_ peaks, such as (015), (1010), and (110) were also observed, indicating that the thin films have multiple orientations. However, these peaks were considerably weaker in thin film with a Te content of 62.0 at%, indicating that this particular thin film with several strong (00 *l*) plane peaks (the highest corresponding to the (0015) plane) was c-axis-oriented and well textured, if not epitaxial. Additional peaks that represent elemental Te were detected with large deviations from the film stoichiometry. Furthermore, in most of our (00 *l*)-oriented thin films, the c-axis may not be perpendicular to the substrate surface, due to the presence of additional non-(00 *l*) peaks.

**Figure 5:**
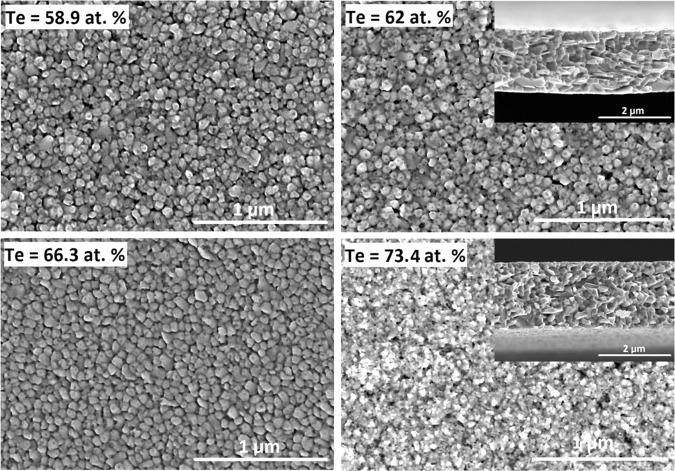
SEM images showing the surface and cross-sectional (inset) morphologies of Sb-Te thin films after thermal annealing.

The effect of substrate temperature during deposition also influences the grain structure and surface morphology of the resulting thin films, as shown in Fig. 5. The grain size and shape, as well as the binding between grain particles, depend significantly on their energy. The grain size of the annealed films is considerably smaller than the grain size of the thin films obtained for film depositions conducted at high substrate temperatures. The microstructure also shows that the grains are dense, uniform, and compact. These observations can be explained by the slow temperature ramp up of 2.5 °C/min during the annealing process; this approach ensured that the as-deposited atoms had sufficient time to diffuse and fill any intervening voids or interspaces to form a continuous thin-film structure during the crystallization process.

The post-annealing microstructural evolution, previously reported for Bi_2_Te_3_ thin films^48^, was considered a consequence of nucleation and new-grain growth of a stable Sb_2_Te_3_ phase from an as-deposited amorphous phase. Furthermore, because the annealing was performed at a lower temperature of 264 °C, the smaller grains did not have sufficient kinetic energy to fuse together to form large-sized grains. Cross-sectional SEM images revealed several layers of grains on the order of the film thickness, thus indicating a slow nucleation process and new layer growth on top of a previously deposited layer. Notably for near stoichiometric thin films having excess Te, grains were observed in the form of layers of platelets, preferentially oriented along the c-axis in which the surface energy would possibly be minimized. In contrast, the thin films with large deviations from stoichiometry assumed a relatively disordered stack configuration of a large number of particle-like grains. These unique, oriented microstructures are expected to favorably influence the electronic and phonon transport in the thin films, as discussed below.

**Figure 6:**
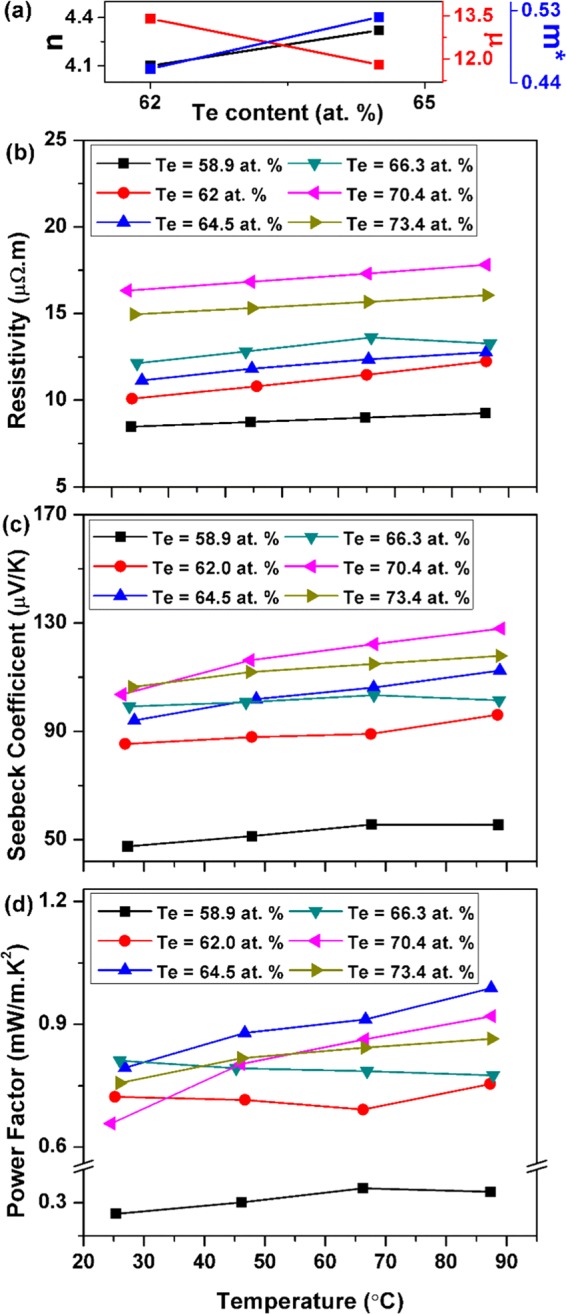
(**a**) Carrier concentration (10^25^ m^−3^), mobility (10^−3^ m^2^/V.s) and DOS effective mass (10^−30^ kg) of the Sb-Te thin films as a function of Te content. Temperature variation of the (**b**) electrical resistivity, (**c**) Seebeck coefficient, and (**d**) PF of annealed Sb-Te thin films.

Figure 6a presents the carrier concentration, mobility and DOS effective mass as a function of Te content and Fig. 6b present the temperature dependence of the films’ electrical resistivity. As the Te content in the thin film increased, the electrical resistivity also increased up to a Te concentration of 70.4 at%. A further increase in Te content resulted in a slight reduction in the electrical resistivity. The electrical resistivity increased linearly with increasing measurement temperature which emphasizes the metallic characteristics in the thin films. Enhanced electronic transport was ensured in the annealed samples, due to their compact and dense structure, lower surface roughness, and fewer number of vacancy defects. This, in turn, significantly increased the carrier mobility which explains the relatively smaller electrical resistivity of the annealed samples (compared with the samples that were deposited at high substrate temperatures). The DOS effective mass, calculated using the Eq. (4), also shows smaller effective mass values when compared to the samples deposited at high substrate temperatures also confirming the above argument. Furthermore, carrier transport in a (00 *l*) oriented and layered structure should not encounter as much interference from interface or grain boundary scattering due to a reduced degree of microstructural randomness. In other words, assuming that significant proportions of the grain boundary interfaces as well as other structural defects in the (00 *l*) layered structure are oriented parallel to the direction of carrier motion, the carrier mobility is expected to increases significantly. These factors should contribute to additional enhancement in the carrier mobility for samples closer to phase stoichiometry. This also explains the measured low electrical resistivity for annealed samples having compositions closer to stoichiometry.

Figure 6c presents the temperature dependence of the Seebeck coefficient. Similar to the electrical resistivity dependence on temperature, the thermopower increased with temperature, as well as with the Te content in the thin film samples. The enhancement in thermopower with the increase in the Te content of the films is attributed to the carrier energy filtering effect due to the energy dependent carrier scattering at Sb_2_Te_3_/Te interface as discussed in the previous section. This was further confirmed by the observed increase in the DOS effective mass of the carrier as the Te content in the sample rises (Fig. 6a). The highest Seebeck coefficient value of 128 μV/K at 90 °C was noted in the case of the thin-film sample with a Te content of 70.4 at%. It would appear that the well-ordered microstructure also has an effect on carrier scattering mechanisms. Assuming that the charge carriers follow classical statistics, the Seebeck coefficient is directly proportional to the carrier scattering parameter according to the following relation^49^:

5 $$S=\pm \frac{{k}_{B}}{e}\left(\frac{5}{2}+s-{\eta }^{\ast }\right)$$

where η* is the reduced Fermi level and s is the scattering parameter. The scattering parameter collectively defines all the scattering mechanisms present within the material. In addition to the impurity/interface scattering due to excess Te atoms, carriers are also scattered by interfaces in a random microstructure, vacancies or interspaces, surface roughness and other structural defects due to deviation from a periodic crystallographic structure. The enhanced carrier mobility that comes with the (00 *l*) layered and ordered crystal structure also entails an overall decrease in carrier scattering processes, as well as a possible reduction in the carrier energy filtering effect in which the fewer number of potential barriers at the grain boundary and defect regions are not sufficient to trap many of the low energy carriers. This may explain why we observed a comparatively lower maximum Seebeck coefficient in the annealed thin films samples. Lastly, the highest value for the Seebeck coefficient was obtained for the thin film with the largest electrical resistivity, due to the combined influence of a relatively high carrier-scattering parameter and a low carrier mobility in off-stoichiometric thin films, as previously discussed.

Figure 6d presents the temperature dependence of the electrical PF, which is calculated by dividing the square of the Seebeck coefficient by the electrical resistivity. The Seebeck coefficient was observed to be highest for the 70.4 at% Te film sample. However, the highest PF of about 1 mW/m ∙ K^2^ was noted for thin film with 64.5 at% Te composition, due to the relatively lower electrical resistivity value of this film. A PF value of 0.9 mW/m ∙ K^2^ was obtained for thin film with a Te content of 70.4 at%. These trends again suggest that deviation from stoichiometry (with excess Te content of about 65 at%) leads to a better performance for Sb_2_Te_3_ thin film. These results also indicate that a higher PF could be obtained if the thin films were synthesized at lower substrate temperatures, followed by annealing, primarily due to the lower electrical resistivity of such samples. This study therefore provides insights into achieving preferential growth orientation in Sb_2_Te_3_ thin films and the favorable effects of the aforementioned special orientation on the TE properties of these thin films.

The thin films prepared using the strategy employed in this work performed well. The largest achievable PF in this work was noted to be either similar to or greater than Sb_2_Te_3_ thin films synthesized using many different deposition techniques as well as bulk material^50,51^. Table 3 shows a comparison of the present work with previous studies for the last 7 years on Sb_2_Te_3_ thin films. As can be observed, the thin film power factor obtained in this study was much better than the Te nanodots embedded electrodeposited Sb_2_Te_3_ thin films^37^ and easily comparable (if not better) to the nanoparticles incorporated Sb_2_Te_3_/Te multilayer thin films deposited by the expensive MBE technique^40^. Notably, the third parameter required for ZT calculations is thermal conductivity, which was not measured in this study. However, the presence of excess Te atoms in film matrix may result in an enhanced phonon scattering at Sb_2_Te_3_/Te interface thereby impeding the thermal transport through the film. As a consequence, the thermal conductivity should reduce leading to further improvement of the ZT.

**Table 3:** Comparison of the literature data for the last 7 years and present work for the Sb-Te thin films.

Ref no., Year	Deposition method	Seebeck coefficient (µV/K)	Power Factor (mW/m.K^2^)
This work	Thermal Evaporation	138	0.87
This work	Thermal Evaporation	112	1
Ref. ^37^., (2013)	Electro deposition	146	0.72
Ref. ^27^., (2015)	Sputtering	145	0.78
Ref. ^31^., (2015)	Thermal Evaporation	112	0.4
Ref. ^40^., (2015)	MBE	150	0.93
Ref .^34^., (2016)	Sputtering	130	0.5
Ref. ^26^., (2017)	Sputtering	153	0.6
Ref. ^28^., (2017)	Sputtering	173	0.1
Ref. ^52^., (2019)	Sputtering	150	0.91
Ref. ^53^., (2019)	Sputtering/Undoped film	400	1.3

The English in this document has been checked by at least two professional editors, both native speakers of English. For a certificate, please see: http://www.textcheck.com/certificate/BKGuHX.

## Conclusion

P-type Sb_2_Te_3_ thin films were successfully grown using vacuum thermal co-evaporation on AlN-coated stainless steel foil. Optimum deposition conditions were determined by evaluating the achieved values of the TE PF. The influences of evaporation rate and substrate temperature on film microstructure, crystal orientation, composition, and transport properties were examined. SEM images and sharp XRD patterns with strong, reduced linewidth peaks indicated polycrystalline thin films with optimum crystalline quality. Columnar thin films with a standard multi-oriented microstructure were obtained when thin films were deposited at a substrate temperature of 300 °C. In contrast, thin films deposited at RT and subsequently annealed at 264 °C had a layered microstructure with a preferential c-axis or (00 *l*) orientation when compositions approached phase stoichiometry. The TE properties were sensitive to the composition. Off-stoichiometric thin films with excess Te content generally had the best TE performance. The best thin film properties were obtained at a measurement temperature of 90 °C, for films deposited on high-temperature substrates (S = 138 µV/K, and ρ = 21.6 µΩ∙m, with a pre-deposited Cr layer) and annealed films (S = 112 µV/K, and ρ = 12.8 µΩ∙m) at 65.0 and 64.5 at% Te content, representing a maximum PF of 0.87 and 1.0 mW/m ∙ K^2^, respectively. In general, we observed a high Seebeck coefficient in the (015)-oriented film and a high electrical conductivity in c-axis-oriented thin films, primarily due to the different scattering mechanisms that the charge carrier experiences. Furthermore, the inclusion of a Cr adhesion layer enhanced the TE properties of the thin films. Thus, this work provides a promising approach for preparing TE thin films, with controlled preferential crystal growth and transport properties for potential application in vertical-type micro-TE devices. The same process can be extended to include a flexible electronic configuration, if the thickness of the stainless steel foil is reduced further.
